# Radiosensitization Induced by Magnetic Hyperthermia of PEGylated Nickel Ferrite Nanoparticles on Breast Cancer Cells

**DOI:** 10.3390/ijms26062706

**Published:** 2025-03-17

**Authors:** Daniele A. Fagundes, Liliam V. Leonel, Luis E. Fernandez-Outon, José D. Ardisson, Raquel G. dos Santos

**Affiliations:** 1 Centro de Desenvolvimento da Tecnologia Nuclear, Avenida Presidente Antônio Carlos, 6627, Belo Horizonte 31270-901, Minas Gerais, Brazil; lvl@cdtn.br (L.V.L.); jdr@cdtn.br (J.D.A.); santosr@cdtn.br (R.G.d.S.); 2 Departamento de Física, Universidade Federal de Minas Gerais, Avenida Presidente Antônio Carlos, 6627, Belo Horizonte 31270-901, Minas Gerais, Brazil; outon@fisica.ufmg.br

**Keywords:** nanobiotechnology, nanostructured ferrites, magnetic induction heating, gamma radiation, thermal radiosensitizers

## Abstract

Magnetic hyperthermia can complement traditional cancer treatments by exploiting the greater heat sensitivity of tumor cells. This approach allows for localized action, increasing its therapeutic effectiveness. In this study, MCF-7 breast cancer cell radiosensitization, induced by the magnetic hyperthermia of PEGylated nickel ferrite magnetic nanoparticles (PEG-NiF MNPs), was evaluated by exposing the cells in the presence of MNPs to an alternating magnetic field followed by ^60^Co gamma irradiation. Superparamagnetic PEG-NiF MNPs (25.6 ± 0.5 nm) synthesized via the hydrothermal method exhibited a hydrodynamic size below 150 nm, a saturation magnetization of 53 emu·g^−1^, biocompatibility of up to 100 µg·mL^−1^, selectivity for breast cancer cells, and an up-to-fivefold increase in therapeutic efficacy of radiation. When combined with magnetic hyperthermia, this increase reached up-to-sevenfold. These results indicate that PEG-NiF MNPs are suitable thermal radiosensitization agents for breast cancer cells.

## 1. Introduction

Despite considerable progress in the development of systems for hyperthermia treatment, intratumoral temperatures achieved with radiofrequency applicators, microwaves, ultrasound, and photothermal therapy are heterogeneous and restricted by target depth and heat dissipation, limiting their clinical efficacy [1,2]. As an alternative, magnetic hyperthermia (MH) using magnetic nanoparticles (MNPs) offers a non-invasive and more localized treatment [3,4].

Although iron oxide MNPs have been used in clinical studies combining magnetic hyperthermia and radiotherapy [5,6], certain aspects of MH still require improvement. The high surface chemical activity leads to oxidation (especially magnetite, due to the high reactivity of Fe^2+^ ion) [7]. The use of spinel ferrites (MFe_2_O_4_; M = bivalent transition metal such as Co^2+^, Ni^2+^, Mn^2+^; Fe = Fe^3+^), represents a strategy to address the problem of chemical stability while maintaining magnetic induction heating properties [8].

Furthermore, the use of MNPs depends on factors such as biodistribution and specific uptake, the presence of biological barriers like the dense extracellular matrix, the intensity of the enhanced permeability and retention (EPR) effect, and their residence time in the bloodstream [9]. The heterogeneity in the MNPs distribution and the difficulty in concentrating them exclusively in the tumor area may compromise the uniformity of heating and increase the risk of adverse effects on healthy tissues, ultimately reducing therapeutic efficiency [10].

In our previous work [11], citrate-coated NiFe_2_O_4_ MNPs (~ 5 nm) were used to radiosensitize breast cancer cells and increase the therapeutic efficacy of radiation. In the present work, NiFe_2_O_4_ MNPs (~ 25 nm) functionalized with PEG (polyethylene glycol bis (3-aminopropyl) terminated) were synthetized to obtain chemically stable and stealthy colloids for additional cell sensitization due to their heat dissipation capacity during MH. Although several studies have highlighted the use of NiFe_2_O_4_ MNPs in drug delivery [12], contrast agent in magnetic resonance imaging [13], antimicrobial activity [14], anti-angiogenic activity [15], and thermotherapeutic applications [16,17], to the best of the authors’ knowledge, this is the first study to evaluate the use of PEGylated NiFe_2_O_4_ MNPs as thermal radiosensitizers for breast cancer cells.

## 2. Results

### 2.1. Nanoparticle Characterization

**Figure 1:**
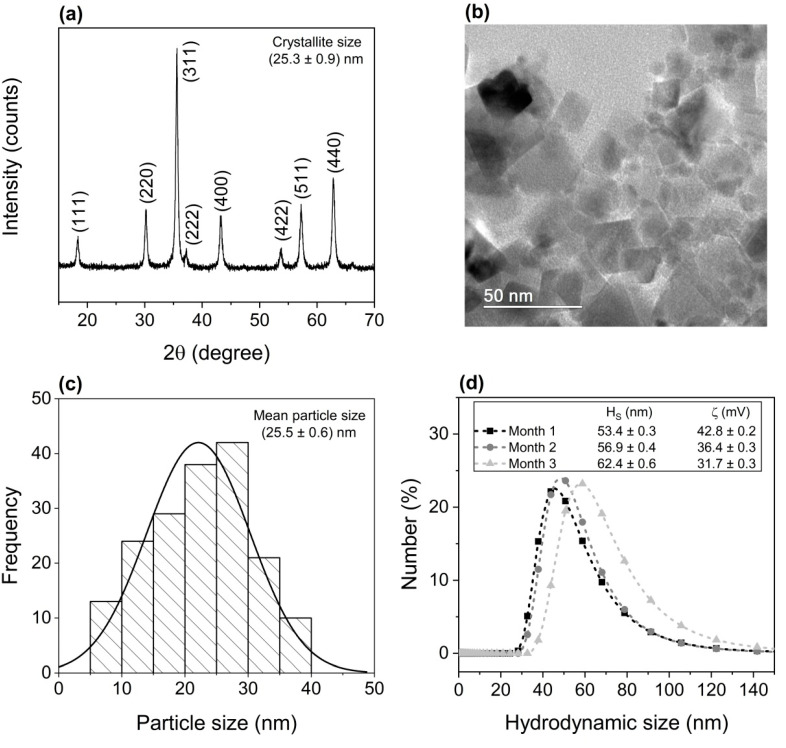
(**a**) X-ray diffraction pattern, (**b**) bright field transmission electron microscopy image, (**c**) NP size distribution, and (**d**) hydrodynamic size distribution over three months of PEG-NiF MNPs. H_S_: hydrodynamic size; ζ: zeta potential.

Figure 1a shows typical diffraction pattern for cubic ferrites, corresponding to planes (111), (220), (311), (222), (400), (422), (511), and (440), confirming the formation of NiFe_2_O_4_ in accordance to Crystallography Open Database (COD#2300289), without secondary phases.

The crystallite size estimated by Scherrer’s equation [18] is (25.3 ± 0.9) nm. This value is compatible with the mean particle size of (25.5 ± 0.6) nm obtained via transmission electron microscopy (TEM) images (Figure 1b,c), suggesting the synthesis of single crystalline MNPs. The monomodal size distribution (Figure 1c) was fitted to a normal curve.

The ^57^Fe Mössbauer spectra of PEG-NiF MNPs obtained at room temperature (RT) and 25 K (Supplementary Figure S1) showed hyperfine parameters (Supplementary Table S1) in accordance with the literature [19,20], confirming the spinel structure observed via X-ray diffraction (XRD) measurements. NiFe_2_O_4_ MNPs PEGylation was evidenced by Fourier transform infrared spectroscopy (FTIR) and thermogravimetric analyses (TGA) (Supplementary Figure S2). The PEG IR spectrum is characterized by a strong band around 1100 cm^−1^, associated with a vibrational mode of C–O–C symmetric stretching [21,22]. This band being slightly shifted in the IR spectrum of PEG-NiF MNPs suggests an interaction between PEG molecules and the surface of MNPs. The band at 597 cm^−1^ corresponds to vibrations of metal–oxygen bonds in tetrahedral sites, further confirming the formation of a spinel ferrite structure, as indicated via XRD and Mössbauer spectroscopy measurements [23]. The TGA curve of PEG-NiF MNPs presents two main mass loss events: the first is attributed to loss of superficial water, and the second is attributed to the thermal decomposition of PEG [24].

The hydrodynamic size distribution, average hydrodynamic size (H_S_), and zeta potential (ζ) of the PEG-NiF MNPs over a three-month period are shown in Figure 1d. Comparison of the average hydrodynamic size with TEM particle size (Figure 1c) suggests the low degree of MNPs aggregation. In addition to PEG steric stabilization, colloidal solutions with zeta potential values larger than 30 mV indicate that the obtained MNPs are electrostatically stabilized (by PEG amine terminations). The trend of increasing hydrodynamic size and reducing zeta potential over time can be associated with the increase in van der Waals and dipolar magnetic interactions due to PEG coating loss.

**Figure 2:**
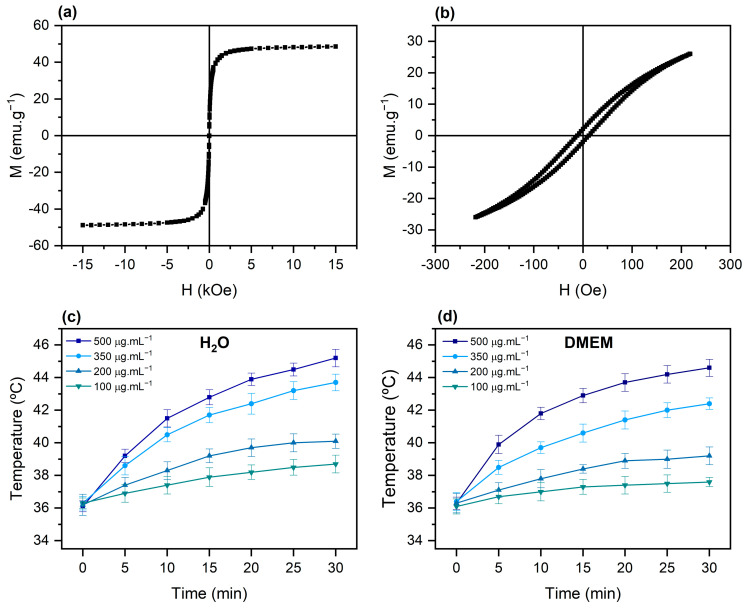
(**a**) Hysteresis curves of PEG-NiF MNPs at RT for a maximum applied magnetic field of 15 kOe and (**b**) 220 Oe. (**c**) Magnetic induction heating curves of PEG-NiF MNPs colloidal solutions in water and (**d**) Dulbecco’s modified eagle medium (DMEM).

Hysteresis curves measured for PEG-NiF MNPs (Figure 2a) indicated superparamagnetic (SPM) behavior at RT, with coercivity and remanent magnetization values close to zero. The value of saturation magnetization (M_S_) at 15 kOe obtained was 48.5 emu·g^−1^. For this determination, the mass of magnetic material was corrected based on TGA analyses (Supplementary Figure S2b). Using the law of approach to saturation, the following M_S_ value was obtained: M_S_ = 49.2 emu·g^−1^. The M_S_ value thus agrees with those obtained by other authors for NiFe_2_O_4_ MNPs of similar size [25].

Figure 2b shows hysteresis curves at a maximum field of 220 Oe, measured with a sweep rate of 1.75 Oe·s^−1^. In this field, PEG-NiF MNPs presented a significant hysteresis area, contrary to what was observed in 15 kOe field. This behavior, also observed by Caetano et al. [26], indicates a contribution to energy loss due to hysteresis in the magnetic induction heating process.

### 2.2. Magnetic Induction Heating of PEGylated NiFe_2_O_4_ Nanoparticles

Magnetic induction heating curves of PEG-NiF MNPs were obtained at different concentrations in water (Figure 2c) and Dulbecco’s modified eagle medium (DMEM) (Figure 2d). When exposed to an alternating magnetic field (220 Oe and 279 kHz), PEG-NiF MNPs with concentrations of 350 and 500 μg·mL^−1^ reached temperatures compatible with application in magnetic hyperthermia (40–45 °C) [27,28] in 30 min.

### 2.3. PEGylated NiFe_2_O_4_ Biocompatibility

**Figure 3:**
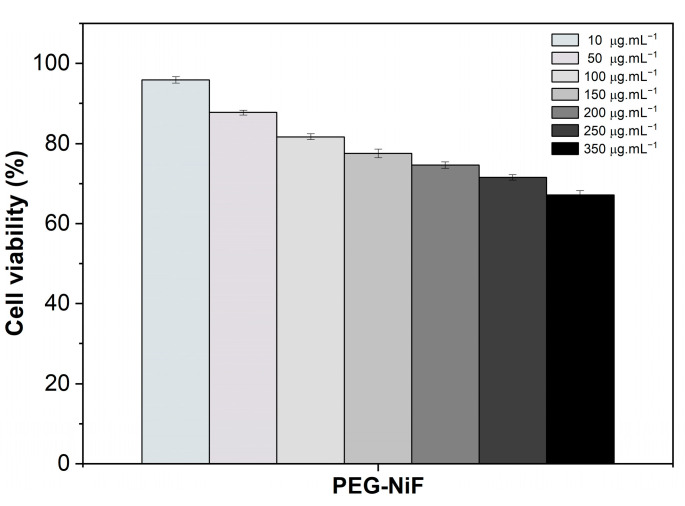
MRC-5 cells viability after 24 h of incubation with different concentrations of PEG-NiF MNPs.

MRC-5 normal fibroblast cells were used as a nontumor cell model to evaluate the biocompatibility of PEG-NiF MNPs. After treatment with MNPs, the majority of the cells presented a density and morphology (fusiform shape and abundant cytoplasm with prolongations) similar to those of the control sample (0 μg·mL^−1^), indicating biocompatibility (Supplementary Figure S3). In fact, PEG-NiF MNPs were biocompatible up to 100 μg·mL^−1^ (Figure 3), exhibiting 80 to 100% of cell viability. Furthermore, even at the highest concentration studied (350 μg·mL^−1^), the viability of PEG-NiF MNPs remained above 65%.

### 2.4. PEG-NiF MNPs Monotherapy

**Figure 4:**
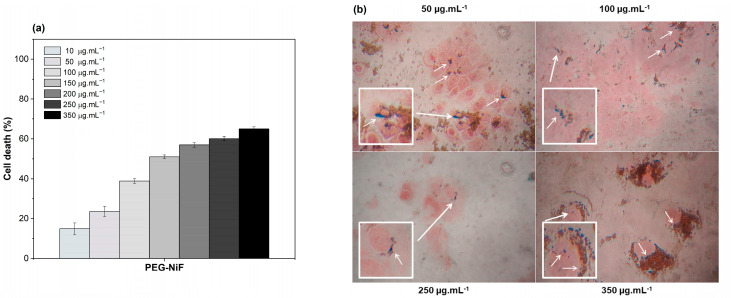
(**a**) Percentage of MCF-7 cells death and (**b**) MCF-7 cellular uptake after 24 h of incubation with different concentrations of PEG-NiF MNPs (Nikon Microscope - 400× magnification; white arrows indicate possible internalization of PEG-NiF MNPs).

MCF-7 breast cancer cells showed significant morphological changes after incubation with MNPs, such as reduced density, formation of cytoplasmic vacuoles, and cell rounding, suggesting cytotoxicity of PEG-NiF MNPs for tumor cells (Supplementary Figure S4). MCF-7 cells’ sensitivity, expressed as a percentage of cell death (Figure 4a), indicated 39% of death at 100 μg·mL^−1^ MNPs concentration, reaching about 65% of death for 350 μg·mL^−1^ concentration (*p* < 0.05).

Figure 4b shows PEG-NiF MNPs uptake by MCF-7 cells. Cell structures were stained red, and Fe-containing MNPs were stained Prussian blue. MNPs can be visualized as blue points nearby cell nucleus and cytoplasm.

### 2.5. Magnetic Hyperthermia Therapy

**Figure 5:**
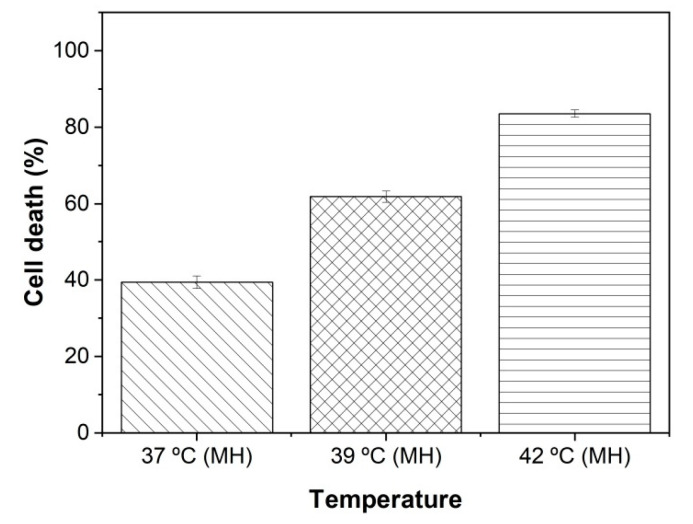
Percentage of MCF-7 cell death after magnetic hyperthermia.

Figure 5 shows the percentage of cell death resulting from cell treatment with magnetic hyperthermia (MH) generated at 220 Oe and 279 kHz via different concentrations of PEG-NiF MNPs: 100 μg·mL^−1^ (37 °C); 200 μg·mL^−1^ (39 °C); 350 μg·mL^−1^ (42 °C).

A characteristic of MNP-mediated hyperthermia is the potential for localized heat generation in tumor regions due to accumulation of NPs [3], sparing normal tissue. As shown in Figure 4b, MCF-7 cells uptake PEG-NiF MNPs. On the other hand, as is shown in Supplementary Figure S3, MRC-5 cells did not uptake PEG-NiF MNPs; then, important heat generation would not be expected in MRC-5 normal cells under magnetic field. Studies suggest that heat stress can induce apoptosis or necrosis depending on the temperature reached [29,30]. Indeed, after MCF-7 cell exposure to an alternating magnetic field (Figure 5), magnetic hyperthermia was induced and evoked significant levels of cell death (*p* < 0.05), validating these MNPs as a hyperthermic agent. The cytotoxic effect was temperature-dependent, reaching approximately 80% of MCF-7 cell death at 42 °C generated by 350 μg·mL^−1^ of PEG-NiF MNPs, while MRC-5 cell viability at this concentration was about 65%, highlighting that its biological application can be safe.

### 2.6. Gamma Radiation Monotherapy (IR)

**Figure 6:**
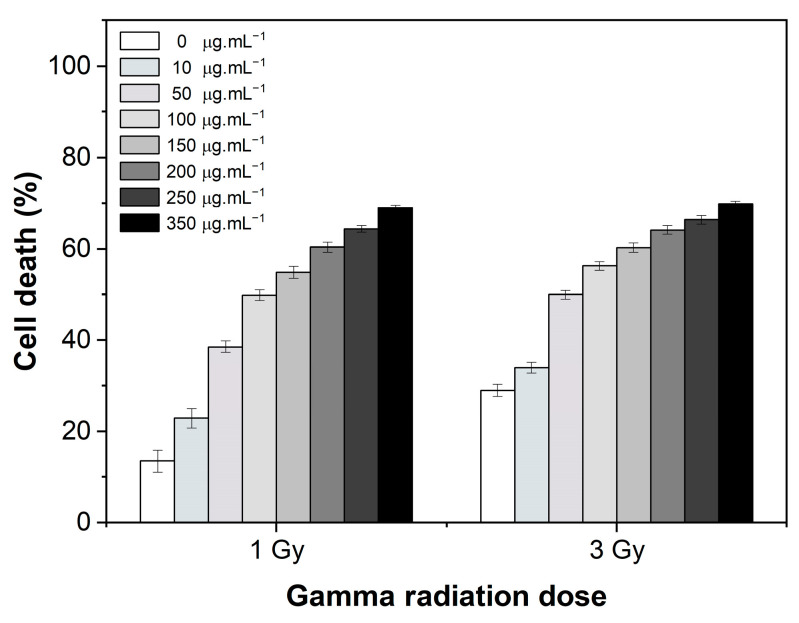
Percentage of MCF-7 cell death after 24 h of combined therapy (MNPs + IR) with PEG-NiF MNPs.

Based on MCF-7 cellular morphology alterations (Supplementary Figure S5) and the percentage of cell death (Figure 6; 0 μg·mL^−1^), gamma radiation monotherapy induced a dose-dependent response (*p* < 0.05), causing a maximum of (29 ± 1)% of cell death at a 3 Gy dose after 24 h of treatment.

### 2.7. Radiosensitizer Potential: MNPs and Gamma Radiation Combined Therapy (MNPs + IR)

The effects of the combined therapy (MNPs + IR), using increasing concentrations of PEG-NiF MNPs and different gamma radiation doses, are shown in Figure 6. The presence of PEG-NiF MNPs induced an increase in the cytotoxic effects of radiation in a dose-dependent manner (*p* < 0.05), indicating a radiosensitizing effect of PEG-NiF MNPs.

The radiation enhancement factor (REF) of the combined therapy (MNPs + IR) (Table 1) indicated an approximate increase of 1.7 to 5.1 times in the therapeutic efficacy of radiation for a 1 Gy dose. The lower radiosensitizing effect for combined therapy with 3 Gy gamma radiation may be due to the fact that the maximum cytolytic effect had already been achieved at this dose.

### 2.8. Thermal Radiosensitizer Potential: MNPs, Magnetic Hyperthermia, and Gamma Radiation Combined Therapy (MNPs + MH + IR)

**Figure 7:**
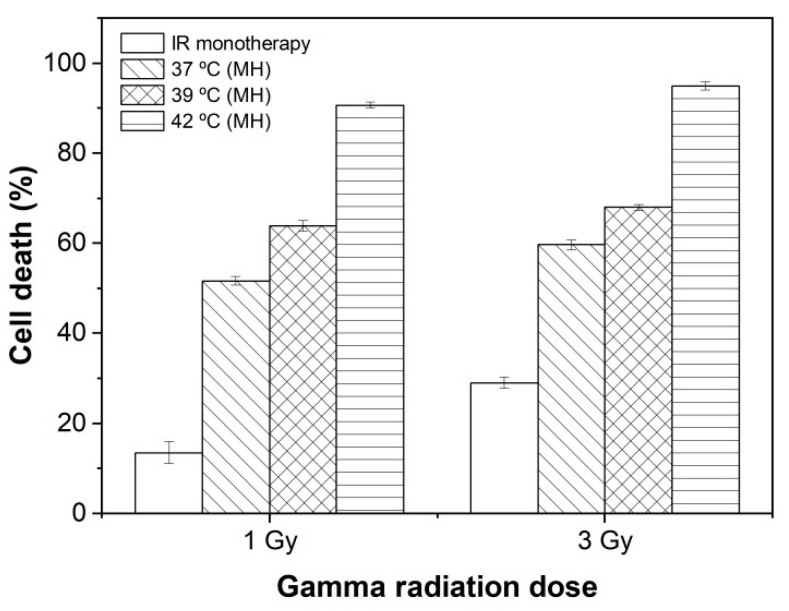
Percentage of MCF-7 cell death after 24 h of combined therapy (MNPs + MH + IR).

Gamma irradiation of cells treated with magnetic hyperthermia in the presence of PEG-NiF MNPs (MNPs + MH + IR) induced higher cell death than radiation monotherapy in a temperature-dependent manner (*p* < 0.001) (Figure 7). This increased effect reached approximately 90% of cell death at 1 Gy combined with 42 °C, validating the PEG-NiF MNPs as thermal radiosensitizing agents. These results are in good agreement with other reports on the combination of hyperthermia mediated by iron oxide MNPs and radiotherapy; however, the PEG-NiF MNPs thermal radiosensitizing effect was superior to those observed in the literature for other MNPs in other tumors [6,31,32,33]. To the authors’ knowledge, this is the first study demonstrating the thermal radiosensitization induced by the magnetic hyperthermia of PEGylated NiFe_2_O_4_ MNPs for breast cancer cells.

Combined treatment of magnetic hyperthermia with 1 Gy gamma radiation (Table 1) resulted in a roughly four- to sevenfold increase in therapeutic efficacy of radiation monotherapy. The combination of MH with 3 Gy, on the other hand, resulted in a lower thermal radiosensitizing effect (around two- to threefold) as the 3 Gy dose already induced a significant amount of death. This inversely proportional dose-dependent behavior was also observed by Johannsen et al. [34].

## 3. Discussion

PEGylated nickel ferrite MNPs produced via the hydrothermal method presented colloidal stability, high crystallinity, and SPM behavior at RT and H_S_ above the renal clearance limit, with low risk of embolism [35,36].

The main mechanisms related to heat generation via the magnetic induction of MNPs are associated with energy loss through hysteresis and energy loss through relaxation (susceptibility loss) [37,38]. The energy dissipated through hysteresis loss is proportional to the area within the hysteresis loop and the frequency. Susceptibility loss is characterized by one or both of the following mechanisms: Néel relaxation, whereby the heat generated from the process of inversion of the particle’s magnetic moment, initially blocked along the easy axis of magnetization, as it aligns with the external magnetic field; and Brownian relaxation, whereby heating results from the friction between the particle and the surrounding medium during the particle’s rotation towards the magnetic field [38]. In this work, a small difference (3%) in heating efficiency in water and DMEM, as shown in Figure 2c,d, suggests a low Brownian relaxation contribution to susceptibility loss. The coercivity exhibited at 220 Oe (Figure 2b) reinforces the probable predominance of hysteresis heating, as it is commonly accepted [39].

PEG-NiF MNPs were biocompatible up to 100 µg·mL^−1^ and induced greater cytotoxic effects in tumor cells compared to normal cells, indicating selectivity for breast cancer (*p* < 0.05). Selective cytotoxic effects may be due to higher ferrite NP uptake to fulfill the iron requirements of malignant cells. Indeed, transferrin receptor (TfR), a membrane protein involved in iron metabolism, has been shown to be upregulated in breast cancer compared to normal breast or fibromas [40,41].

It was observed that MH induced greater cell death than MNPs monotherapy. It is worth noting that the heat generated (37 °C) by the MH at the MNPs lowest concentration (100 µg·mL^−1^), equivalent to physiological temperature, resulted in approximately 40% cell death. According to Liu et al. [42], experimental evidence indicates that MNPs produce nanoscale heat effects without macroscopic temperature increase. Other authors [43,44] report that even though a macroscopic temperature increase is not noticeable, a slight local temperature increase near the MNPs surface can damage the cell membrane or organelles, resulting in the release of cathepsins from lysosomes, causing apoptosis or direct cell death. At higher MNPs concentrations, the MH effect was enhanced, leading to approximately four times more cell death than MNPs monotherapy. Heat can cause the denaturation of cell proteins, compromise plasma membrane integrity, alter cytoskeleton organization, and interfere in the mitochondrial structure and function, leading to tumor cell death via apoptosis or necrosis [45,46].

The up-to-fivefold increase in the cytotoxic response of combined therapy (MNPs + IR) when compared to radiation monotherapy can be attributed to the absorption of ionizing radiation and the transfer of electromagnetic energy from the incident photon to the MNPs via Compton, photoelectric, or pair production interactions, as previously discussed by Fagundes et al. [11] and Mansouri et al. [47]. This results in local dose deposition around NPs, which is intensified by the secondary diffusion of energy from the MNPs, associated with the electron cascade effect, depending on initial photon energy. Additionally, the presence of iron can catalyze Haber–Weiss and Fenton reactions, enhancing the effectiveness of radiotherapy through the production of reactive oxygen species [48,49].

Thermal radiosensitization up-to-sevenfold provided by the combined therapy (MNPs + MH + IR) can be attributed to the effect of magnetic hyperthermia, leading to the modulation of proteins involved in processes such as cell remodeling, DNA repair, autophagy, and apoptosis [50,51], associated with the radiation effect due to the ability of ferrite MNPs to absorb ionizing radiation, emit secondary electrons, and act as a catalyst for Haber–Weiss and Fenton reactions [11,46,47].

## 4. Materials and Methods

### 4.1. Synthesis of PEGylated NiFe_2_O_4_ Nanoparticles

NiFe_2_O_4_ MNPs were synthesized via the hydrothermal method. Briefly, stoichiometric quantities of nickel (II) nitrate hexahydrate (>98%) and iron (III) nitrate nonahydrate (>98%) were dissolved in 15 mL of water. Nitrate solution was dripped into a NaOH solution (30 mL, 1.2 M) under stirring. The mixture (pH 10) was transferred to a Teflon vessel and sealed in a stainless steel autoclave. The temperature was maintained at 130 °C (2 bar pressure) for 2 h. After the reaction time, the precipitate was washed with water until pH 7 and dried at 100 °C for 4 h. For functionalization with PEG (polyethylene glycol bis (3-aminopropyl) terminated) (Mn ~ 1500), 20 mg of NiFe_2_O_4_ MNPs were dispersed in 19.5 mL of water in an ultrasonic bath for 60 min. After this time, 0.5 mL of a PEG solution (1.6 mg·mL^−1^) was added. This mixture remained in ultrasonic bath for 90 min. The solution with PEGylated MNPs (PEG-NiF) was subjected to dialysis for 24 h on a cellulose membrane (cutoff: 12–14 kDa). All reagents were of analytical grade, purchased from Sigma-Aldrich (St. Louis, MO, USA), and used without any additional purification. Aqueous solutions were prepared utilizing water from a Milli-Q water purification system (Millipore, Burlington, MA, USA).

### 4.2. Nanoparticle Characterization

XRD measurements of ferrite power samples were acquired in the angular range (2θ) of 10° to 70° using an Ultima IV diffractometer system (Rigaku, Wilmington, MA, USA) with Cu Kα radiation (λ = 1.54059 Å) and a scanning velocity of 1° min^−1^. TEM images of MNPs solutions dripped on a carbon-coated copper grid were obtained with a Tecnai G2-20—FEI SuperTwin microscope (Hillsboro, OR, USA), operating at 200 kV. ^57^Fe Mössbauer spectra in transmission mode were obtained using a ^57^Co/Rh source calibrated with reference to α-Fe at room temperature and 25 K, without the application of an external magnetic field. Infrared spectra were measured using KBr discs in a Nicolet 6700 Fourier transform infrared spectrometer (Thermo Fisher Scientific, Waltham, MA, USA) through 64 scans in the wavenumber range from 4000 to 400 cm^−1^ with a resolution of 4 cm^−1^. TGA were carried out using a DTG-60H (Shimadzu, Chiyoda, Tokyo, Japan) in a temperature range from 25 to 800 °C, with a heating rate of 10 °C·min^−1^ and synthetic air atmosphere with a flow of 100 mL·min^−1^. Samples were diluted in Milli-Q water at a concentration of approximately 250 µg·mL^−1^ and manually homogenized. PEGylated MNPs hydrodynamic size and zeta potential were determined using a Zetasizer Nano ZS 3000 HSA equipment (Malvern Panalytical, Malvern, Worcestershire, UK) at 25 °C, having 173° detection optics and a 532 nm laser. Hysteresis loops were measured using a vibrating sample magnetometer model 7404 (Lake Shore Cryotronics, Westerville, OH, USA) at RT with a maximum applied field of 15,000 and 220 Oe. Magnetic induction heating tests were carried out with a 220 Oe and 279 kHz alternating magnetic field, generated by an EasyHeat 0224 variable magnetic field inductor (Ambrell, Rochester, NY, USA) coupled to a three-turn copper coil.

### 4.3. Cell Culture

Normal human lung fibroblast (MRC-5) and human breast cancer (MCF-7) cells, obtained from the American Type Culture Collection (Manassas, VA, USA), were cultured as a monolayer in Dulbecco’s modified eagle medium (DMEM) supplemented with 10% fetal bovine serum and 1% penicillin/streptomycin in a water-jacketed incubator at 37 °C in a 5% CO_2_ atmosphere. Cells with 80% confluence were subcultured with trypsin. All treatments were performed in 24- or 96-well plates with exponentially growing cells.

### 4.4. Cell Treatment

Six cell groups were used: (1) control; (2) MNPs monotherapy (MNPs); (3) gamma radiation monotherapy (IR); and combined therapies: (4) MNPs and gamma radiation (MNPs + IR); (5) MNPs and magnetic hyperthermia (MNPs + MH); and (6) MNPs, magnetic hyperthermia, and gamma radiation (MNPs + MH + IR).

In group 1 (control), the cells were incubated only with the culture medium. In group 2 (MNPs), cells were treated with different PEG-NiF MNPs concentrations (10–350 μg·mL^−1^). In group 3 (IR), high dose rate (HDR) radiation of 95 Gy.h^−1^ was applied to cells in two doses: 1 and 3 Gy. Irradiations were carried out in multipurpose panoramic irradiator, equipped with a dry-stored ^60^Co source (2200 TBq—Gammacell Eγ_1_ = 1.17 MeV; and Eγ_2_ = 1.33 MeV). In group 4 (MNPs + IR), cells were pre-treated with PEG-NiF MNPs and, after 2 h of incubation, were irradiated. In group 5 (MNPs + MH), cells were resuspended in DMEM and, in the presence of PEG-NiF MNPs concentrations of 100, 200, or 350 μg·mL^−1^, were subjected to magnetic hyperthermia for 1 h. Afterwards, cells were seeded in culture plates with 50 μg·mL^−1^ MNPs concentration in each well. In group 6 (MNPs + MH + IR), the cells received MNPs + MH pre-treatment, and 24 h later, they were submitted to gamma radiation.

The effect of each procedure was evaluated via cell morphology and viability assays.

### 4.5. Cell Viability Assay

Biocompatibility and cytotoxicity of PEG-NiF MNPs were determined by measuring the reduction of MTT (3-(4,5-dimethyl-2-thiazolyl)-2,5-diphenyl-2H-tetrazolium bromide) reagent in formazan crystals [52]. After each corresponding treatment, cells were stained with MTT (0.5 mg·mL^−1^) according to the manufacturer’s protocol. Formazan crystals were solubilized with dimethyl sulfoxide, and samples’ absorbance at 570 nm were measured using a UV–visible microplate reader.

The percentage of cell viability was normalized in relation to control cells. The results of the therapies combined with gamma radiation were expressed as a radiation enhancement factor (REF) calculated according to Equation (1).

(1) $$REF=\frac{\%\;of\;cell\;death\;due\;to\;combined\;therapy}{\%\;of\;cell\;death\;due\;to\;gamma\;radiation\;monotherapy}$$

### 4.6. Statistical Analysis

Data statistical evaluation was performed using analysis of variance (ANOVA) followed by the Bonferroni’s test for multiple comparisons (Prisma GraphPad software, version 5.01). Differences with a *p*-value ≤ 0.05 were considered statistically significant. Results were expressed as mean ± standard error of the mean.

### 4.7. Morphological Alterations and Nanoparticle Cellular Uptake

Cells’ morphological alterations and MNPs’ cellular uptake were evaluated via optical microscopy. After treatment, cells were stained with Prussian blue and neutral red, as previously described in Fagundes et al. [11]. Briefly, the cells were washed with phosphate-buffered saline (PBS, pH 7.4) and fixed with absolute methanol. After removing the methanol, a 1:1 mixture of 2% potassium (II) ferrocyanide trihydrate and 4% hydrochloric acid (in PBS) was added, and the cells were incubated, protected from light. After 30 min, cells were washed with PBS, stained with 0.1% neutral red solution, and washed again. Images were captured using a Nikon Eclipse TS100 microscope (Shinagawa, Tokyo, Japan) coupled to a Nikon D3000 camera (Sathorn, Bangkok, Thailand).

## 5. Conclusions

Given the current challenges in radiotherapy for cancer treatment, including the need to reduce adverse effects and improve therapeutic efficacy, efforts have focused on utilizing magnetic nanoparticles as hyperthermic and radiosensitizing agents. In this work, superparamagnetic nickel ferrite nanoparticles prepared via the hydrothermal method and functionalized with PEG were biocompatible with MRC-5 normal fibroblast cells up to a concentration of 100 μg·mL^−1^. The combination of PEG-NiF MNPs and gamma radiation (^60^Co) at a dose of 1 Gy in MCF-7 breast cancer cells resulted in an up-to-fivefold increase in therapeutic efficacy compared to radiation monotherapy. The use of magnetic induction heating as an adjuvant enhanced the effect of radiation monotherapy up-to-sevenfold. These results indicate that PEG-NiF MNPs are suitable candidates for the development of thermal radiosensitization agents for breast cancer cells.

## Figures and Tables

**Table 1:** Radiation enhancement factor for the combined therapy (MNPs + IR) and (MNPs + MH + IR).

Gamma Radiation Dose	Combined Therapy (MNPs + IR)							Combined Therapy (MNPs + MH + IR)		
	Concentration of PEG-NiF MNPs (µg·mL^−1^)							MH Temperature (°C)		
	10	50	100	150	200	250	350	37	39	42
1 Gy	1.7	2.9	3.7	4.1	4.5	4.8	5.1	4.4	5.0	7.0
3 Gy	1.2	1.7	1.9	2.1	2.2	2.3	2.4	2.1	2.3	3.3

## Data Availability

The raw data supporting the conclusions of this article will be made available by the authors on request.

## Supplementary Materials

The following supporting information can be downloaded at https://www.mdpi.com/article/10.3390/ijms26062706/s1.
