# Supplementary material for: Radiosensitization Induced by Magnetic Hyperthermia of PEGylated Nickel Ferrite Nanoparticles on Breast Cancer Cells
Source: Int J Mol Sci. 2025 Mar 17;26(6):2706. doi: 10.3390/ijms26062706 (PMC11942335; doi:10.3390/ijms26062706)

Supplementary Materials

Radiosensitization Induced by Magnetic Hyperthermia of Pegylated Nickel Ferrite Nanoparticles on Breast Cancer Cells

Contents:

1. Supplementary Figure S1 – ^{57}Fe Mössbauer spectra of PEG-NiF MNPs at room temperature and 25 K (Mössbauer spectra were fitted with two sextets associated to Fe^{3+} in tetrahedral (A) and octahedral (B) sites. The third additional sextet (red line) is related to the presence of particles that exhibit superparamagnetic (SPM) behavior at RT. At 25 K the effect of thermal fluctuations decreased, promoting magnetic ordering of the MNPs).

2. Supplementary Table S1 - Isomer shift (δ), quadrupole displacement (ε), hyperfine magnetic field (Bhf) and relative spectral area of PEG-NiF MNPs at room temperature (RT) and 25 K.

3. Supplementary Figure S2 – (a) Infrared spectra and (b) thermogravimetric curves of nickel ferrite magnetic nanoparticles before (NiF) and after (PEG-NiF) PEGylation.

4. Supplementary Figure S3 – MRC-5 cells morphology after 24 hours of incubation with different concentrations of PEG-NiF MNPs (Nikon Microscope - 400 x magnification; Prussian blue and neutral red staining; formation of cytoplasmic vacuoles [↑] and cell rounding [*]).

5. Supplementary Figure S4 - MCF-7 cells morphology after 24 hours of incubation with different concentrations of PEG-NiF MNPs (Nikon Microscope - 400 x magnification; Prussian blue and neutral red staining; formation of cytoplasmic vacuoles [↑] and cell rounding [*]).

6. Supplementary Figure S5 - MCF-7 cells morphology after 24 hours of radiation monotherapy (Nikon Microscope - 400 x magnification; Prussian blue and neutral red staining; the formation of cytoplasmic vacuoles [↑] and cell rounding [*] is indicated).

Supplementary Figure S1. ^{57}Fe Mössbauer spectra of PEG-NiF MNPs at room temperature and 25 K (Mössbauer spectra were fitted with two sextets associated to Fe^{3+} in tetrahedral (A) and octahedral (B) sites. The third additional sextet (red line) is related to the presence of particles that exhibit superparamagnetic (SPM) behavior at RT. At 25 K the effect of thermal fluctuations decreased, promoting magnetic ordering of the MNPs).

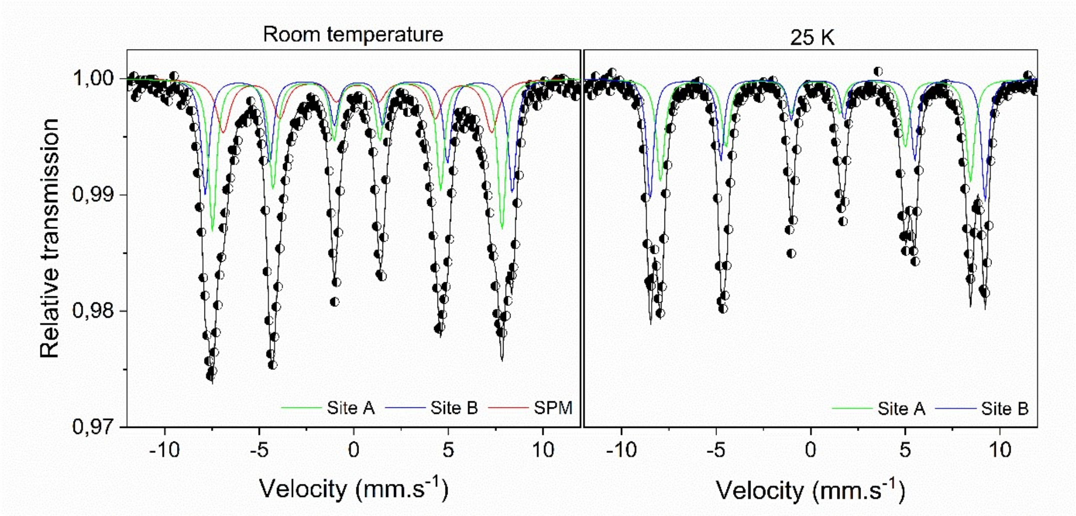

Supplementary Table S1. Isomer shift (δ), quadrupole displacement (ε), hyperfine magnetic field (Bhf) and relative spectral area of PEG-NiF MNPs at room temperature (RT) and 25 K

**Sample**	**Sites**	**δ (±0.05)** (mm.s^{-1})	**ε (±0.05)** (mm.s^{-1})	**B_{hf} (±0.5)** (T)	**Area (±1)** (%)
PEG-NiF **(RT)**	A	0.29	0.00	44.0	36
	B	0.35	0.01	50.4	28
	Sextet (SPM)	0.27	0.01	47.5	36
PEG-NiF **(25 K)**	A	0.35	0.00	50.8	55
	B	0.46	0.00	54.9	45

Supplementary Figure S2. (a) Infrared spectra and (b) thermogravimetric curves of nickel ferrite magnetic nanoparticles before (NiF) and after (PEG-NiF) PEGylation.

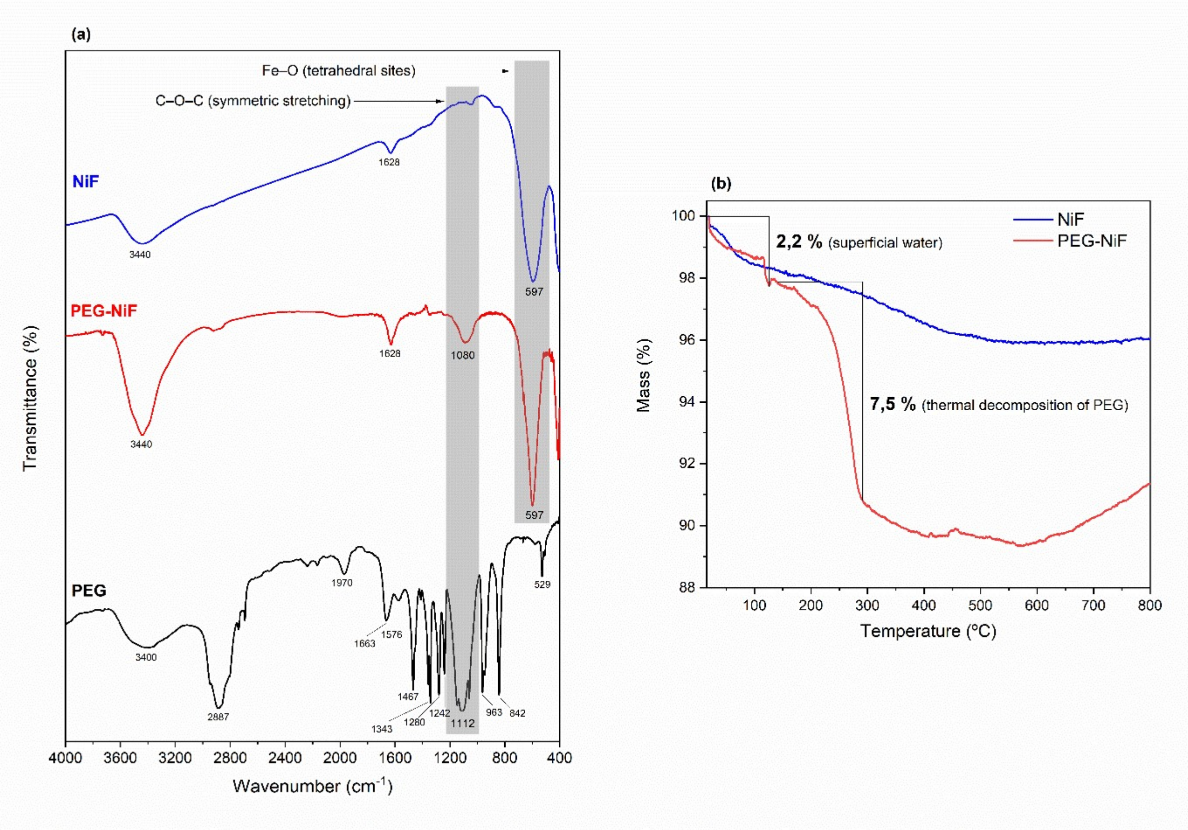

Supplementary Figure S3. MRC-5 cells morphology after 24 hours of incubation with different concentrations of PEG-NiF MNPs (Nikon Microscope - 400 x magnification; Prussian blue and neutral red staining; formation of cytoplasmic vacuoles [↑] and cell rounding [*]).

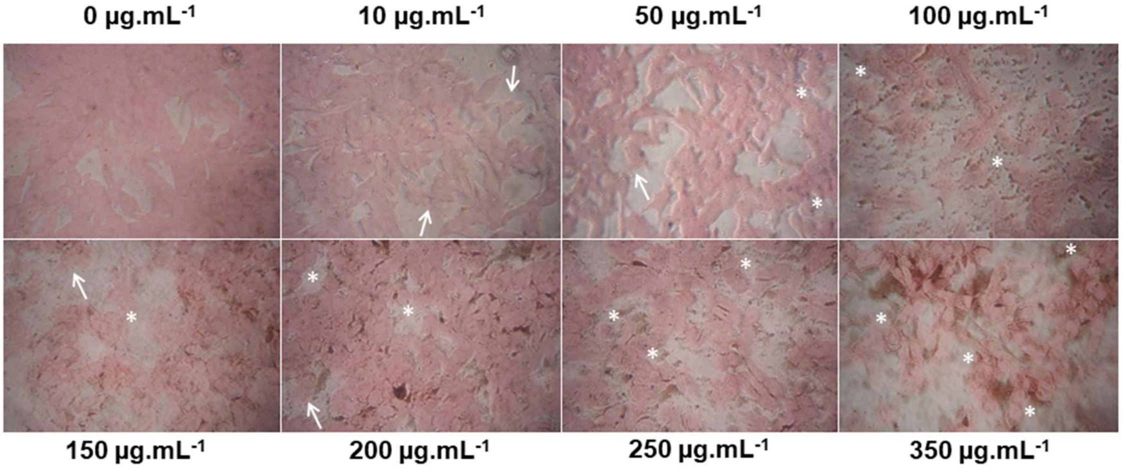

Supplementary Figure S4. MCF-7 cells morphology after 24 hours of incubation with different concentrations of PEG-NiF MNPs (Nikon Microscope - 400 x magnification; Prussian blue and neutral red staining; formation of cytoplasmic vacuoles [↑] and cell rounding [*]).

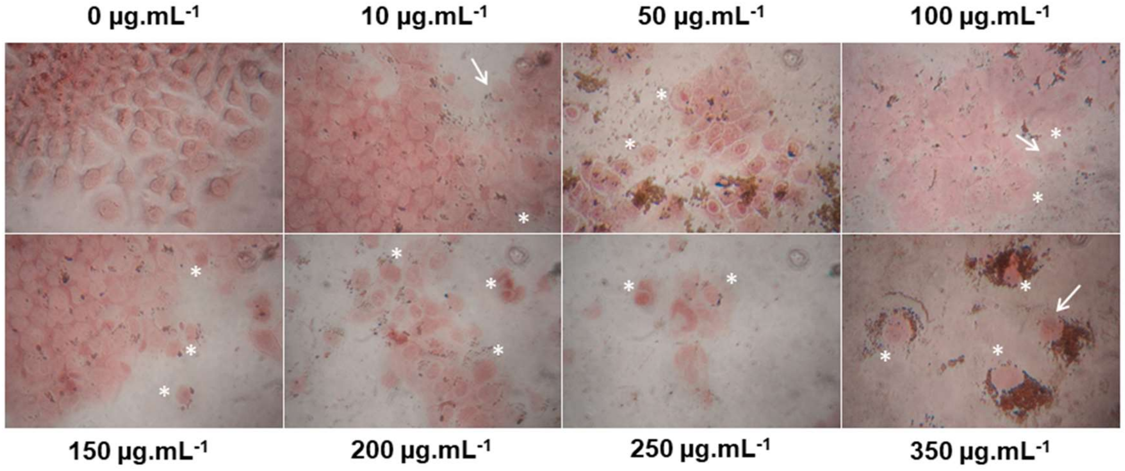

Supplementary Figure S5. MCF-7 cells morphology after 24 hours of radiation monotherapy (Nikon Microscope - 400 x magnification; Prussian blue and neutral red staining; the formation of cytoplasmic vacuoles [↑] and cell rounding [*] is indicated).

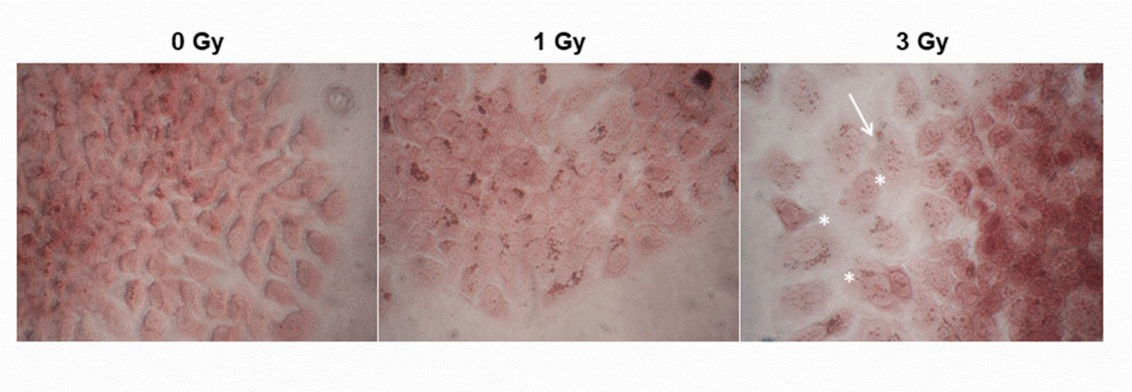

Supplement: Supplementary file 1 [file ijms-26-02706-s001.zip › ijms-3408397-supplementary.pdf]
